# Lexical diversity in Parkinson’s disease

**DOI:** 10.1186/s40734-015-0017-4

**Published:** 2015-03-02

**Authors:** Charles Ellis, Yolanda F Holt, Thomas West

**Affiliations:** Department of Communication Sciences and Disorders, East Carolina University, 3310H Health Sciences Building, MS 668, Greenville, NC 27834 USA

**Keywords:** Parkinson’s disease, Basal ganglia, Discourse, Language

## Abstract

**Background:**

Parkinson’s disease (PD) is a neurodegenerative syndrome of the basal ganglia (BG) believed to disrupt cortical-subcortical pathways critical to motor, cognitive and expressive language function. Recent studies have shown subtle deficits in expressive language performance among individuals with PD even in the earliest stage of the disease. The objective of this study was to use measures of lexical diversity to examine expressive language performance during discourse production in a sample of individuals with PD.

**Methods:**

Twelve individuals with idiopathic Parkinson’s disease (PD) were compared to twelve matched, neurologically intact controls on measures of lexical diversity. Three minute discourse samples describing a typical day were collected and analyzed for lexical diversity with the CHILDES program using measures of type token ratio (TTR) and voc-D (*D*).

**Results:**

Comparisons of three minute discourse samples indicated non-significant differences between individuals with PD and controls in word productivity (387 vs 356; p = .48). Similarly, there were also non-significant differences on measures of lexical diversity between the two groups (TTR = .45 vs.44; p = .50 and *D* 74 vs 68; p = .23).

**Conclusions:**

These results suggest that lexical diversity during discourse production among individuals with PD is similar to non-neurological controls. These findings indicate that lexical diversity is an aspect of expressive language performance that is not impacted by the disease process in the earliest stages.

## Background

Parkinson’s disease (PD) is a neurodegenerative syndrome most often associated with reductions in motor performance. In the United States, 50,000-60,000 new cases are diagnosed annually [1]. The disease process associated with PD centers on the basal ganglia, however the disease progressions courses through multiple systems affecting the brainstem and eventually affecting the cerebral cortex [2]. In addition to motor deficits, many individuals with PD experience changes in cognitive and language skills. Declines in motor performance are readily detectable in PD and correlate with reported neuropathological stages of PD [2]. In contrast, although expressive language deficits have been identified in PD, they are not reported as frequently as more commonly observed motor speech deficits.

The basis of hypothesized expressive language production deficits in PD emerges from models of basal ganglia (BG) function which indicate critical connections between the BG and other areas of the brain. More specifically, the BG are connected to the cerebral cortex via a collection of cortical-BG-thalamic-cortical circuits that vary in function [3,4]. These connections offer support for an anatomical basis for expected deficits in expressive language which is primarily governed by the cerebral cortex [5,6]. Using these models of BG function in PD, studies of language production in PD have identified expressive language performance deficits. For example two reviews of expressive language in PD noted morphosyntactic, lexical semantic and language production breakdowns as linguistic complexity increased [7,8].

Language and other cognitive deficits are not as easily identifiable until later in the PD disease process. However, they too, appear to develop gradually and concurrently with the neuropathological stages of the disease beginning with the cortical-BG-thalamic-cortical circuits connecting subcortical structures to motor areas [2-4,9,10]. The disease process is then believed to disrupt cortical-BG-thalamic-cortical circuits subsequently diminishing non-motor connections to the cerebral cortex, particularly the frontal lobes which are vital to cognitive performance.

It has been hypothesized that in addition to early motor symptoms, subtle cognitive declines that are not severe enough to justify a diagnosis of dementia can be present (i.e. at the onset of initial motor symptoms) [9,10]. It is tenable then that impairments in expressive language performance may be a specific example of declines in cognitive performance in PD that are more difficult to detect. The relative influence of PD on expressive language performance in PD has yet to be adequately examined. To test the hypothesis of early cognitive declines in PD, novel diagnostic measures sensitive to subtle changes in cognitive performance on skills such as expressive language in PD are required.

Discourse analyses have been suggested as a method to characterize expressive language performance deficits in a range of neurological diseases [11,12]. According to Fergadiotis & Wright, discourse analyses allow researchers to observe complex cognitive/linguistic behaviors during a common form of communication therefore offering a functional analysis of language skills [13]. Discourse is a complex goal directed activity requiring intent, planning and task persistence (i.e. executive function). Discourse production represents the highest level of expressive language use or language procedures designed to serially assemble complex utterances determined by context and a specific goal [14,15]. Discourse is a dynamic cognitive process comprised of microlinguistic (language features that occurs within sentences) and macrolinguistic (language features that that crosses sentence borders) levels of organization [16]. Consequently, any compromise of this dynamic process may result in impaired discourse production that is independent of coexisting motor speech difficulty [15].

Discourse production has been previously examined in PD [17,18]. However, conclusions drawn were based on studies that included participants with more advance disease stages or did not consider specific language deficits in favor of concomitant cognitive and speech impairments. To address these issues the objective of this study was to examine a specific language outcome, lexical diversity (LD), in a sample of individuals with PD to determine if LD is influenced by PD early in the disease process. The rationale for examining lexical diversity emerges from studies that suggest disruptions in how language is used occurs in PD. For example, Holtgraves and colleagues found that individuals with PD exhibited more “under-informativeness” than non-PD controls during interviews. Under-informativeness or too little information provided was hypothesized as the result of decreased executive control, mental status and speech comprehension. Similarly, because Rogers and colleagues observed executive deficits in PD patients, we believe the temporal aspect of discourse may result in differences when compared to those without PD [19]. Consequently, we hypothesized that lexical diversity, a microlinguistic feature that occurs during discourse production might offer additional insights into the contributions of PD to disruptions in expressive language performance.

LD is defined as “a range of vocabulary deployed in a text by a speaker that reflects his/her capacity to access and retrieve target words from a relatively intact knowledge base i.e., lexicon for the construction of higher linguistic units (p.1415) [13]. It is believed that LD depends on word frequency and the interaction of phonologic, semantic and syntactic language subsystems [13]. Measures of LD are well documented in the child language literature. The most basic measure of LD is the number of different words (NDW) in a sample calculated as a division of the number of different words by the total number of words in the sample. NDW is significantly influenced by sample length and individuals who generate more verbal output exhibit higher levels of LD [20].

The most commonly used measure of LD is the type-token ratio (TTR). TTR is the ratio of the total number of different words to the total number of words in the sample. TTRs that are closer to zero are an indication of limited vocabulary diversity whereas values closer to one reflect greater LD or more diverse vocabulary use. Similar to NDW, TTR is also sensitive to sample length in that as the sample increases the probability of producing new words decreases and the TTR decreases [20]. Consequently, comparisons between speakers who produce samples varying in length are confounded by the length of the samples that are produced.

A third measure that has emerged and developed to address issues related to sample length experienced with the use of NDW and TTR is voc-*D.* voc-D (*D*) is a an estimate of LD derived from a combination of an algebraic transformation model and curve fitting. *D* allows a more accurate comparison of LD in discourse samples because it does not rely on sample length [21]. *D* can be calculated in discourse samples using the voc-D program in Computerized Language Analysis (CLAN). *D* has been previously used as a measure of lexical diversity in individuals with neurogenic communication disorders such as aphasia [13,20,22].

The purpose of this study was to use discourse in individuals with PD and non-neurological controls to examine the influence of PD on LD. Discourse production requires an integration of multiple cognitive skills including: linguistic organization, linguistic planning and working memory, which is sensitive to neurological disease [15]. We hypothesized that an analysis of discourse would provide samples of sufficient length to evaluate LD in PD where dementia was not a contributing factor. We sought to test the hypothesis that individuals with PD would have less LD when compared to matched non-neurologically impaired controls. We examined subjects classified in Hoehn & Yahr (H&Y) stages II and III which are individuals with bilateral involvement yet are absent of significant motor impairment and are physically independent [23]. Individuals at these stages tend to have limited reductions in overall communication ability due to motor declines relative to more advance disease stages. Thus, we wanted to ensure that speech production issues would not mask overall expressive language performance and subsequently measures of lexical diversity. Therefore, it was expected that the measures of LD in individuals with PD would not be related to any reductions in motor speech performance.

## Method

### Description of the subjects

Participants consisted of 12 community dwelling individuals diagnosed with idiopathic PD (hereafter referred to as experimental subjects) by a movement disorders neurologist using the strict criteria of the UK Brain Bank [24] and 12 individuals who were age, education, ethnicity and gender matched and neurologically intact (hereafter referred to as control subjects). All participants were recruited from the North Florida/South Georgia Veterans Health System. The study was approved by the University of Florida IRB and VA Research and Development Committee and all participants gave written informed consent. All participants were male, right handed, and had no history of prior stroke, dementia, brain tumor, or head trauma. All had at least a seventh grade education, functional hearing for normal conversation, functional vision for reading tasks, spoke English as their primary language, and demonstrated expressive language skills within intact range for normal conversation. Functional hearing and expressive language was determined by the first author (CE) a certified and licensed speech-language pathologist. All subjects (experimental and control) exhibited scores of 26 or better on the Mini Mental Status Exam (MMSE) [25].

Each experimental subject presented with a minimum of 3 of 4 cardinal features of PD (resting tremor, rigidity, bradykinesea, postural instability) and had no history of deep brain stimulation or brain lesion therapy. The parkinsonism of each experimental subject was rated with the Hoehn & Yahr (H&Y) Staging Scale for PD and classified by predominate feature (tremor vs. rigidity) [23].

#### Standardized assessments

The Boston Naming Test (BNT) [26] and Wechsler Memory Scale – Logical Memory I (WMS-LMI) [27] were administered to examine potential group differences relative to language form (BNT) and the influence of short term memory on language form and use (WMS-LMI).

#### Discourse data collection

Discourse samples were collected from experimental subjects by the first author in their homes prior to their first daily dose of anti-parkinsonian medication (levadopa, dopamine agonists, amantadine, and/or selegiline). The duration of time since their last dose was at least 12 hours. Collecting samples prior to their first daily dose of anti-parkinsonian medication ensured they were in their “off” medication state to maximize dopamine depletion, a major putative cause of cognitive dysfunction in PD. Five of the 12 experiemental subjects were newly diagnosed with PD and had no history of PD medication use at the time of the study. Control subjects were also examined primarily in their homes.

All subjects were instructed to discuss a typical day for a minimum of three minutes. In the event that subjects stopped before 3-minutes, a standardized verbal cue (“Tell me more about that”) was provided to continue the narrative until the 3-minute minimum was achieved. A Sony VN-480 PC digital voice recorder was used to record each subject’s samples. The investigator provided the subjects instructions for each sample followed by a restatement of the topic. Audio-taping began at the point when the topic was restated.

#### Motor speech performance ratings

After completion of data collection, the motor speech performance of all subjects was rated. An independent judge (certified and licensed speech language pathologist) blind to the neurological status of the subjects rated each audio sample. Each sample was rated on a 5-point scale of speech intelligibility [28]. Ratings ranged from 1 (no detectable disorder) to 5 (no functional speech).

### Motor speech ratings reliability

#### Transcription and segmentation

The first three minutes of all language samples were transcribed verbatim by a professional transcription service. Each sample was divided into communication units (CU), defined as the shortest allowable independent clause and related dependent clauses [29]. Individual CU’s were defined primarily by syntax, however prosodic and semantic features were used at times when the unit could not be determined entirely by syntax. All unintelligible words were excluded from the analysis. In instances where the location of coordinating conjunctions such as “and”, “but” and “or” was unclear, their prosodic feature determined their final location at the beginning or ending of the communication unit. One-word responses were not considered in the communication unit calculation.

#### CU reliability

Three trained raters participated in the project to establish reliability for identification of CU’s. Raters were blinded to the neurological status of subjects that generated the samples used for the analyses. One trained rater analyzed 100% of the samples that were used for the analysis. Two additional trained raters independently analyzed 15% of the total sample. Intra-class correlation coefficients (ICC) were calculated by using a two-way mixed model with repeated measures to evaluate scoring agreement among the raters for CU’s. The ICC score for words was .99.

### Computerized analysis of discourse language variables

#### Sample preparation and calculation of LD

The first author entered the transcribed samples into the CHILDES CLAN program using the CHAT format specified in the Tools for Analyzing Talk – Electronic Edition [30]. The Mac-based CLAN program was used on a Macbook Pro computer. In brief summary, samples were entered with emphasis on content words (i.e. nouns, verbs, adjectives and adverbs). Repetitions, repairs and fillers were not entered and thereby excluded from analysis. To estimate lexical diversity the CLAN “voc-*D*” function was used which generates total number words and two measures of LD; D and TTR.

#### LD reliability

The third author entered 15% of randomly selected samples into the CLAN program using the same CHAT format. Measures of lexical diversity were calculated independently for comparisons. Simple correlations of TTR and *D* were calculated as a measure of reliability. Correlations of .91 for TTR and .96 for *D* were achieved for each measure indicating a high agreement.

### Statistical analysis

For group comparisons, independent samples t-tests were conducted for continuous variables and Chi-square for categorical variable with the criterion for significance set at p < .05 for all variables.

## Results

### Demographic comparisons

Table 1 lists demographic, cognitive and language comparisons for subjects in the study. Two-tailed t-test (p < .05), revealed non-significant differences between the two groups for age, education, short term memory (WMS-LMI), and language form (BNT) and general cognitive ability (MMSE).

**Table 1:** **Demographic, cognitive, and language comparisons for PD and control subjects**

	**PD subjects**		**Controls**		
**Variable**	**M**	**SD**	**M**	**SD**	**p**
Age	71.8	13.2	72.6	13.5	.89
Education	12.0	1.3	12.8	2.8	.36
Parkinson years	3.6	4.6			
H & Y stage	2.4	.5			
BNT	52.8	6.7	51.8	8.4	.75
MMSE	28.6	1.4	28.8	1.7	.80
WMS-LMI	27.5	11.5	30.6	14.4	.57

Values are means ± S.D. *p* values are derived from comparisons of PD subjects to normal controls. Parkinson Years = the number of years since PD subjects were initially diagnosed with PD. H & Y = Hoehn and Yahr; BNT = Boston Naming Test, all items administered; MMSE = Mini Mental Status Exam; WMS-LMI = Wechsler Memory Scale – Logical Memory I subtest.

### Motor speech performance ratings

Group comparisons revealed a significant difference between the PD group (M = 2.2, SD .72) and control group (M = 1.3, SD .62) on intelligibility ratings, (X^2^ = 10.7; p = .003). Scores ranged from 1–3 for each group [1 (no detectable disorder), 2 (obvious speech disorder with intelligible speech), and 3 (reduction in speech intelligibility)].

### Computerized analysis of lexical diversity

Table 2 list measures of word productivity and lexical diversity. Two-tailed t-test (p < .05), revealed non-significant differences between the two groups on the number of words produced (PD = 387 vs controls = 357; p = .48). No significant differences were made on measures of TTR (PD TTR = .45 vs controls TTR = .44; p = .50) and *D* (PD *D* = 74 vs controls *D* = 68; p = .23).

**Table 2:** **Group performances on measures of lexical diversity**

	**PD subjects**		**Controls**		
**Variable**	**M**	**SD**	**M**	**SD**	**p**
Words	356.8	77.5	387.0	120.9	.48
TTR	.45	.04	.44	.05	.50
*D*	73.8	12.6	67.5	12.3	.23

Values are means ± S.D. *p* values are derived from comparisons of PD subjects to normal controls.

## Discussion and conclusions

The results of this study did not support the hypothesis that individuals with PD would exhibit less LD during discourse production when compared to matched non-neurological controls. Comparisons to non-neurologically impaired control subjects did not yield statistically significant differences. Although reductions in lexical diversity have been observed in other neurological populations who experience language deficits, we found that individuals with PD exhibited very similar lexical diversity whether measured with TTR or D. These findings are important because they add to current lines of research which indicate expressive language issues in PD a disorders primarily related to motor deficits. Although this analysis did not yield groups differences and support recent studies that suggest disruptions in language skills exist early in PD, it does support the current literature that suggests specific deficits are related “language use” issues rather than “language structure” issues (word and sentence productivity, syntax, grammaticality, etc.). For example, in previous work, we found that although individuals with PD did not differ from controls on measures of language structure in discourse (narrative productivity, communication units, and number of cohesive ties produced), they did differ on measures of cohesive adequacy [31]. These data are also supported by studies of language pragmatics or the use of verbal and non-verbal social communication among individuals with PD [19].

These preliminary findings may suggest that LD is a measure that may not be sensitive to changes in PD. It is possible that LD measures lack the sensitivity to differentiate changes in expressive language in patients early on in PD. The lack of observed differences may alternately suggest fronto-basal ganglia disruptions that influence linguistic processing for expressive language do not occur in the earliest H&Y stages of PD. We expected that the temporal aspects of discourse production would elicit group differences. This hypothesis is based on findings by Rogers and colleagues that report executive deficits in patients functioning at H & Y stages I & II [32]. Therefore, our results suggest that even though cognitive skills may be affected in PD populations, H & Y stages I-III may not be associated with the level of neuropathologic disease required to negatively influence expressive language performance.

It is also possible that other features of discourse production (i.e. cohesion and coherence) may be more sensitive to PD and probably should be considered in future studies. Similarly, the literature related to language performance in PD suggests that measures of language structure (word and sentence productivity, syntax, grammaticality, etc.) have failed to consistently differentiate PD from normal language performance. Therefore, some propose that measures of language use (language pragmatics) may be more sensitive to language related in PD [33,34].

The non-significant findings in light of recent hypotheses of earlier cognitive deficits in PD highlight two specific issues. First, although the neuropathological progression of PD has been described extensively, the exact impact of disease progression on cognitive skills such as language remains unclear. Braak and colleagues propose that individuals with PD may progress through a phase similar to mild cognitive impairment (MCI) prior to overt dementia [9]. However, it is important to note that the disease progression described by Braak and colleagues does not correlate specifically with clinical disease staging using the H&Y scale. Therefore, the cognitive changes that occur in patients with PD/MCI and the changes that occur during the transition from MCI to overt dementia are unclear. Consequently, difficulty exists in attempts to distinguish the level of cognitive ability across the continuum of the two cognitive disorders. Second, the impact of cognitive deterioration in PD on expressive language and other cognitive skills is unknown.

A minor secondary finding of this study was although there were differences in motor speech performance, word productivity (number of words produced) did not differ between the two groups. On average, the participants with PD produced a greater number of words over the course of three minutes. We considered that the greater but non-significant difference in words produced may have been a function of the greater number of cues required among individuals with PD (45 vs 18) to elicit the three minute samples. However, because the focus of this study was measures of LD and LD is primarily a reflection of the range of words produced rather than the total, the increased need for verbal cuing likely did not influence the results reported here.

Future studies should be designed to evaluate individuals at all disease stages as well as equivalent representation of tremor and rigid predominant features would provide additional information about influence of PD disease progression on expressive language. It would additionally be better to divide patients for clinical studies by disease duration rather than stage as a majority of all patients are in stage II and III. We also acknowledge that alternate explanations such as reduced attention, depression, medication state, and apathy should be measured and correlated with changes in discourse production. However, the results of this study offer a number of future research possibilities that will increase our understanding of the influence of PD on expressive language production. Comparisons of PD and other basal ganglia diseases would help differentiate language disruptions that may occur. A detailed examination of all possible ways expressive language can be impaired following disease will be required to clarify the influences of PD on expressive language.

## References

[1] National Parkinson Foundation. 2014. http://www.parkinson.org/parkinson-s-disease.aspx. Accessed on September 3, 2014

[2] Braak H, Del Tredici K, Rub U, de Vos RA, Jansen Steur EN, Braak E (2003). Staging of brain pathology related to sporadic Parkinson's disease. Neurobiol Aging.

[3] Middleton FA, Strick PL (2000). Basal ganglia and cerebellar loops: motor and cognitive circuits. Brain Res Brain Res Rev.

[4] Middleton FA, Strick PL (2000). Basal ganglia output and cognition: evidence from anatomical, behavioral, and clinical studies. Brain Cogn.

[5] Alexander MP, Stuss DT, Knight (2002). Disorders of language after frontal lobe injury: evidence for the neural mechanisms of assembling language. Principles of frontal lobe function.

[6] Salmon DP, Heindel WC, Hamilton JM, Litcher DG, Cummings JL (2001). Cognitive abilities mediated by frontal-subcortical circuits. Frontal-subcortical circuits in psychiatric and neurological disorders.

[7] Murray L (2008). Language and Parkinson's disease. Annu Rev Appl Linguist.

[8] Altmann LJ, Troche MS (2011). High-level language production in parkinson's disease: a review, Parkinson's Disease.

[9] Braak H, Rub U, Jansen Steur EN, Del Tredici K, de Vos RA (2005). Cognitive status correlates with neuropathologic stage in Parkinson disease. Neurology.

[10] Braak H, Rub U, Del Tredici K (2006). Cognitive decline correlates with neuropathological stage in Parkinson's disease. J Neurol Sci.

[11] Glosser G, Deser T (1991). Patterns of discourse production among neurological patients with fluent language disorders. Brain Lang.

[12] Wilson BM, Proctor A (2000). Oral and written discourse in adolescents with closed head injury. Brain Cogn.

[13] Fergadiotis G, Wright HH (2011). Lexical diversity for adults with and without aphasia across discourse elicitation tasks. Aphasiology.

[14] Alexander MP (2006). Impairments of procedures for implementing complex language are due to disruption of frontal attention processes. J Int Neuropsychol Soc.

[15] Ash S, Moore P, Antani S, McCawley G, Work M, Grossman M (2006). Trying to tell a tale: discourse impairments in progressive aphasia and frontotemporal dementia. Neurology.

[16] Marini A, Carlomagno S, Caltagirone C, Nocentini U (2005). The role played by the right hemisphere in the organization of complex textual structures. Brain Lang.

[17] Illes J, Metter EJ, Hanson WR, Iritani S (1988). Language production in Parkinson's disease: acoustic and linguistic considerations. Brain Lang.

[18] Murray LL (2000). Spoken language production in Huntington's and Parkinson's diseases. J Speech Lang Hear Res.

[19] Holtgraves T, Fogle K, Marsh L (2013). Pragmatic language production deficits in parkinson's disease. Adv Park Dis.

[20] Wright HH, Silverman SW, Newhoff M (2003). Measures of lexical diversity in aphasia. Aphasiology.

[21] McKee G, Malvern D, Richards B (2000). Measuring vocabulary using dedicated software. Literacy Linguist Comput.

[22] Fergadiotis G, Wrigh HH, West T (2013). Measuring lexical diversity in narrative discourse of people with aphasia. Am J Speech Lang Pathol.

[23] Hoehn MH, Yahr MD (1967). Parkinsonism: onset, progression and mortality. Neurology.

[24] Bower JH, Maraganore DM, McDonnell SK, Rocca WA (2000). Influence of strict, intermediate, and broad diagnostic criteria on the age- and sex-specific incidence of Parkinson's disease. Mov Disord.

[25] Folstein MF, Folstein SE, McHugh PR (1975). "Mini-mental state". A practical method for grading the cognitive state of patients for the clinician. J Psychiatr Res.

[26] Kaplan E, Goodglass H, Wientraub S (1983). Boston naming test scoring booklet.

[27] Wechsler D (1997). Wechsler Memory Scale III.

[28] Yorkston KM, Beukelman DR, Strand EA, Bell KR (1999). Management of Motor Speech Disorders in Children and Adults.

[29] Hunt KW (1965). Grammatical structures written at three grade levels. (Research Report No. 3).

[30] MacWhinney B (2000). The CHILDES project: Tools for analyzing talk, Vol 1: Transcription format and programs.

[31] Ellis C, Okun MS, Gonzalez-Rothi LJ, Crosson B, Rogalski Y, Rosenbek JC (2006). Expressive language use after PD: deficits in use but not form. Mov Disord.

[32] Rogers RD, Sahakian BJ, Hodges JR, Polkey CE, Kennard C, Robbins TW (1998). Dissociating executive mechanisms of task control following frontal lobe damage and Parkinson's disease. Brain.

[33] Hall D, Ouyang B, Lonnquist E, Newcombe J (2011). Pragmatic communication is impaired in Parkinson disease. Int J Neurol.

[34] McNamara P, Durso R (2003). Pragmatic communication skills in patients with Parkinson's disease. Brain Lang.

